# An Uncommon Occipital Horn Arachnoid Cyst in a Newborn

**DOI:** 10.7759/cureus.110803

**Published:** 2026-06-13

**Authors:** Rachel Battersby, Rasha Alnajjar, Jacquelyn Garcia, Brian Shafter, Thiagarajan Nandhagopal

**Affiliations:** 1 Family Medicine, Western University of Health Sciences, Pomona, USA; 2 Pediatrics, Kern Medical Center, Bakersfield, USA; 3 Family Medicine, Clinica Sierra Vista, Bakersfield, USA

**Keywords:** arachnoid cyst, neonatal, occipital horn, prenatal imaging, ventricular dilation

## Abstract

This is a case of a full-term male newborn with the rare diagnosis of an arachnoid cyst that is located in the left occipital horn of the lateral ventricle. Prenatal ultrasounds demonstrated progressive dilation of the left lateral ventricle without additional anomalies. On day two of life, an ultrasound confirmed moderate to marked dilation of the left occipital horn. Magnetic resonance imaging revealed a dilated left occipital horn containing a well-circumscribed, cystic lesion with cerebrospinal fluid measuring 4.3×2.1 cm, consistent with an arachnoid cyst. Pediatric neurosurgery was consulted and recommended further evaluation and follow-up. This case highlights an unusual cyst location within the left occipital horn of the lateral ventricle in a newborn. Furthermore, it underscores the importance of early diagnosis and imaging to guide management.

## Introduction

Arachnoid cysts are benign, cerebrospinal fluid-filled sacs that account for about 1% of intracranial lesions in children [1]. Their exact etiology is uncertain, though they are believed to result from an abnormal splitting of the arachnoid membrane during embryogenesis [1]. In some cases, histopathologic findings such as inflammatory cells or hemosiderin deposits suggest an inflammatory or traumatic origin [1]. Most arachnoid cysts are congenital, originating during fetal development and caused by abnormal meningeal and brain development [1]. Arachnoid cysts are generally theorized to develop from abnormalities in cerebrospinal fluid (CSF) flow, which, due to pulse pressure, could dissect the meninges and form an enclosed sac [2]. Most arachnoid cysts are detected incidentally on neuroimaging for other reasons or during prenatal scans. They may present at any age, but clinically significant cysts tend to present in pediatric patients with a male predominance of 2:1 [3].

Most arachnoid cysts occur in the anterior or middle cranial fossae and the retrocerebellar cistern [1]. In contrast, intraventricular arachnoid cysts are exceedingly rare, with only a few cases reported [4]. These may originate from the arachnoid layer within the choroidal fissure [4]. Those intraventricular cysts are of significance as they could rarely obstruct the ventricular outflow tract, further causing neurologic symptoms or hydrocephalus [3].

In a retrospective descriptive review of cysts discovered during prenatal ultrasounds and a cohort study comparing patients with additional ultrasound abnormalities to those with isolated findings, it was found that cysts with sizes greater than 2 cm on the second-trimester ultrasound and location outside of the interhemispheric fissure may indicate the need for further evaluation and eventual fenestration [5]. Detecting such lesions during prenatal screening is vital to ensure that the neonate gets adequate treatment promptly and to reduce any possible long-term or life-threatening effects of the cyst.

Here, we present a rare case of a newborn with an arachnoid cyst measuring 4.3 cm×2.1 cm located in the left occipital horn of the lateral ventricle.

## Case presentation

A male infant was born at 38 weeks and one day gestation via normal spontaneous vaginal delivery to a gravida 2, para 1 mother (G2P1, now P2) following an uncomplicated pregnancy. Prenatal ultrasonography performed by maternal-fetal medicine demonstrated progressive left lateral ventricular dilation, measuring 1.7 cm at 28 weeks and six days gestation and increasing to 2.2 cm by 32 weeks and six days. No additional fetal anomalies were identified. The infant’s head circumference at birth measured 36.0 cm, corresponding to approximately the 90th percentile for gestational age and sex.

A cranial ultrasound performed on day 2 of life to follow-up on prenatal findings demonstrated moderate-to-marked dilation of the left occipital horn of the lateral ventricle (see Figure 1). Pediatric neurosurgery at Valley Children's Hospital (VCH) was consulted and recommended further evaluation with magnetic resonance imaging (MRI). MRI revealed dilation of the left occipital horn containing a well-circumscribed cerebrospinal fluid (CSF)-intensity cystic lesion measuring 4.3×2.1 cm, consistent with an arachnoid cyst (see Figures 2, 3). Given the cyst's size, the patient’s young age, and the lesion's rarity, the patient was referred to a pediatric neurosurgical specialist for further evaluation and management. 

**Figure 1:**
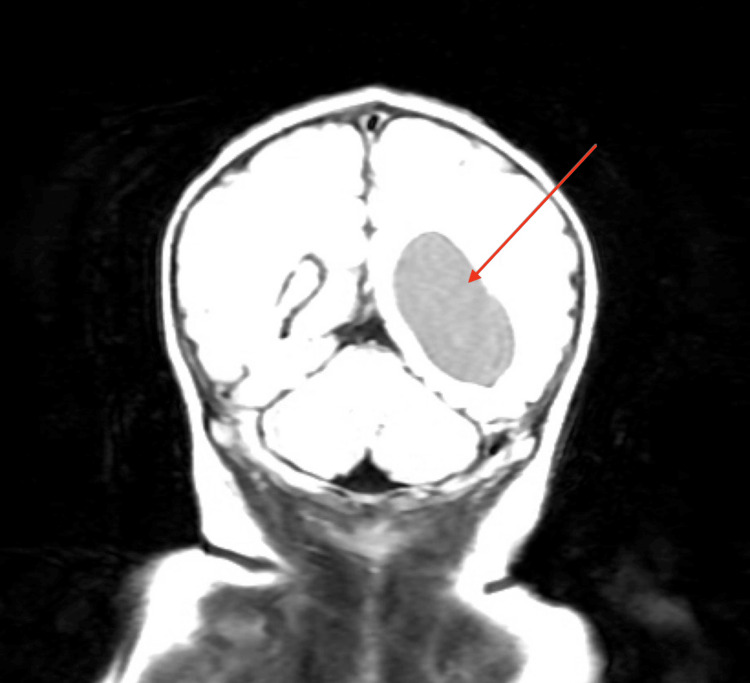
Coronal T2 fluid-attenuated inversion recovery (FLAIR) MRI image

**Figure 2:**
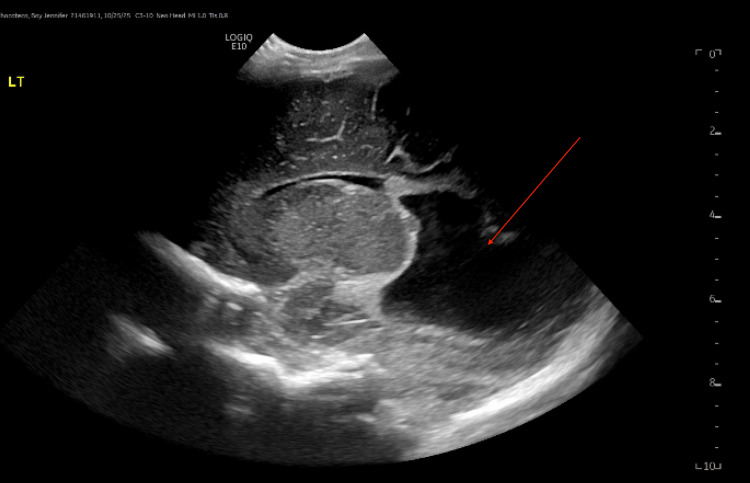
Ultrasound demonstrating moderate to marked dilation, primarily of the left occipital horn

**Figure 3:**
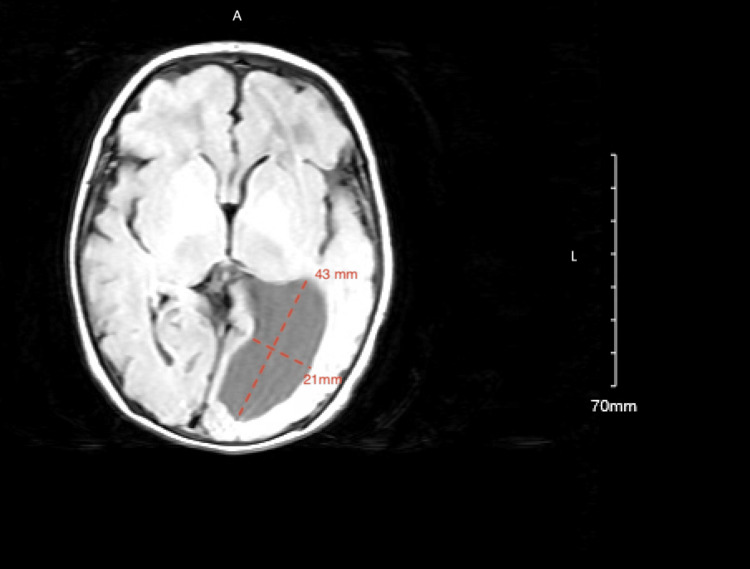
Axial T2 fluid-attenuated inversion recovery (FLAIR) image demonstrating a cyst within the left occipital horn that measures 4.3x2.1 cm in size.

At 12 weeks of age, a head ultrasound was completed, demonstrating no enlargement of the right lateral, third, or fourth ventricles. The posterior horn of the left lateral ventricle was enlarged in the parieto‑occipital region. The finding was favored to represent encephalomalacia, similar to prior MRI. A small left choroid plexus cyst measuring 3×2×3 mm was also noted. No germinal matrix, intraventricular, or intraparenchymal hemorrhage was identified, and the visualized extra‑axial spaces and imaged portions of the superior sagittal sinus were normal.

The patient currently remains under the care of an outside neurosurgery team with ongoing serial imaging surveillance. No surgical intervention has been indicated to date. A review of the available follow-up records demonstrated that the patient has continued to feed well and has remained clinically stable without reported neurologic concerns.

The possible differential diagnoses for the large fluid-filled intracranial space seen on MRI included arachnoid cyst, porencephalic cyst, and cystic hygroma. Given the lesion’s location and imaging findings, continued close monitoring for signs and symptoms of hydrocephalus or other neurologic abnormalities was essential. Throughout the hospital course, the patient’s vital signs remained within normal limits. A comprehensive neurologic evaluation was unable to be performed due to the patient’s age; however, no acute abnormalities were reported. Long-term clinical and radiographic outcomes are unknown, as subsequent follow-up occurred with specialists at an outside facility.

## Discussion

Arachnoid cysts are typically discovered incidentally during prenatal screening or during evaluation for macrocephaly, developmental delay, or seizures. This case describes a neonate with an incidental left occipital horn arachnoid cyst identified during prenatal ultrasonography with maternal-fetal medicine. Following birth, the infant remained asymptomatic, and the cyst was managed conservatively with neurosurgical follow-up and serial imaging surveillance.

Most arachnoid cysts occur in the middle cranial fossa, particularly in the temporal region, followed by the posterior fossa and suprasellar cistern [1]. Other cystic lesions may also be encountered within the lateral ventricles in neonates, including frontal horn cysts (FHCs), which are relatively uncommon. FHCs are typically located adjacent to the anterior horns and demonstrate a characteristic thin-walled, elliptical morphology on ultrasound. Although reported in up to approximately 0.5-0.9% of neonates, they are generally considered benign, transient findings that resolve spontaneously within the first months of life and are not associated with significant mass effect or progressive ventricular dilation [6]. In contrast, arachnoid cysts located within the lateral ventricles are exceedingly rare, with only a few cases documented in the literature [4]. These cysts are thought to arise from invagination of the arachnoid membrane along the choroidal fissure during development [1]. Unlike FHCs, which are typically incidental and self-limited, the lesion in this case behaved as a true space-occupying process, raising concern for mass effect and necessitating close postnatal evaluation [6]. Furthermore, because of their intraventricular location, they can occasionally mimic other cystic lesions such as neuroepithelial cysts, porencephalic cysts, or ependymal cysts, making neuroimaging essential for accurate diagnosis.

The natural history of arachnoid cysts is not extensively documented in the literature. In a cohort study with a mean follow-up of 3.5 years involving 111 patients, most arachnoid cysts remained stable, while 11 increased in size and 13 decreased. Younger age at presentation was significantly associated with cyst enlargement and a greater likelihood of requiring surgery (p=0.001). No patients older than four years at presentation experienced cyst enlargement or required surgical intervention [7].

The current management of asymptomatic arachnoid cysts tends to be conservative, emphasizing observation and serial neuroimaging unless there is evidence of mass effect, hydrocephalus, or neurological compromise [7,8]. The role of prophylactic surgery remains questionable in asymptomatic patients [9]. On the other hand, in symptomatic cases, surgical intervention may be warranted.

It is important to note that this case report does have several limitations. The most significant is that the entirety of long-term clinical and radiographic follow-up is not available due to the patient's subsequent transfer of care to an outside facility. As a result, long-term neurologic development, cyst management, and overall clinical outcomes could not be fully assessed. Additionally, because intraventricular arachnoid cysts in neonates are exceedingly rare, conclusions regarding clinical progression and optimal management remain limited. Further case reports and longer-term follow-up studies are needed to understand the clinical course and management of such cysts in infants.

## Conclusions

This case is noteworthy because of the cyst’s unusual location within the left occipital horn of the lateral ventricle in a newborn. Only a handful of such cases have been reported, and most are diagnosed later in infancy or childhood. Recognition of this lesion type is important to avoid misdiagnosis and unnecessary intervention. In our patient, the lesion was detected prenatally as progressive unilateral ventricular dilation and later confirmed postnatally by MRI as an arachnoid cyst. Given the cyst’s increasingly large size and rare location in this neonate, close follow-up and further evaluation by a pediatric neurosurgeon are critical. However, because the patient’s subsequent care occurred at an outside facility, long-term clinical and radiographic outcomes are unavailable at this time. This limits our ability to assess progression, neurodevelopmental outcomes, need for future intervention, and broader conclusions regarding clinical management.
